# Syndemic conditions predict lower levels of physical activity among African American men who have sex with men: A prospective survey study

**DOI:** 10.1371/journal.pone.0213439

**Published:** 2019-03-13

**Authors:** Jingwen Zhang, Ann O’Leary, John B. Jemmott, Larry D. Icard, Scott E. Rutledge

**Affiliations:** 1 Department of Communication, University of California, Davis, Davis, California, United States of America; 2 Centers for Disease Control and Prevention, Atlanta, Georgia, United States of America; 3 Annenberg School for Communication and Department of Psychiatry of Perelman School of Medicine, University of Pennsylvania, Philadelphia, Pennsylvania, United States of America; 4 School of Social Work, Temple University, Philadelphia, Pennsylvania, United States of America; Burapha University, THAILAND

## Abstract

African American men are disproportionately affected by, not only HIV/AIDS, but also chronic non-communicable diseases. Despite the known benefits of physical activity for reducing chronic non-communicable diseases, scant research has identified factors that may influence physical activity in this population. A growing literature centers on the syndemic theory, the notion that multiple adverse conditions interact synergistically, contributing to excess morbidity. This secondary data analysis examined two primary questions: whether syndemic conditions prospectively predicted physical activity, and whether, consistent with the syndemic theory, syndemic conditions interacted to predict physical activity. Participants were 595 African American men who have sex with men (MSM), a population underrepresented in health research, enrolled in a health-promotion intervention trial from 2008–2011. We used generalized-estimating-equations models to test the associations of syndemic conditions and resilience factors measured pre-intervention to self-reported physical activity 6 and 12 months post-intervention. As hypothesized, reporting more syndemic conditions pre-intervention predicted reporting less physical activity 6 and 12 months post-intervention, adjusting for the intervention. However, contrary to the syndemic theory, we did not find evidence for the interaction effects of syndemic conditions in predicting physical activity. Receiving high school education and having greater social network diversity predicted more physical activity whereas older age predicted less physical activity. To our knowledge, this is the first study to examine the syndemic theory in relation to physical activity. Although reporting a greater number of syndemic conditions was related to reduced physical activity, there was no evidence for synergy among syndemic conditions.

## Introduction

After decades of efforts to reduce health disparities, African American men continue to have the highest mortality rate among all racial/ethnic and gender groups in the United States [1–3]. African American men are disproportionately affected by not only HIV/AIDS [4], but also chronic non-communicable diseases (NCDs) such as cardiovascular disease, stroke, hypertension [5], diabetes mellitus [6], and obesity [7] relative to other men. For instance, African American men ages 30–39 years are about 14 times more likely to develop kidney failure due to hypertension than are White men [8]. Furthermore, African American men are 60% more likely to die from stroke and 30% more likely to die from heart disease than are white men [9, 10].

African American men who have sex with men (MSM), the group accounting for the largest number of African Americans with HIV/AIDS [4], has received most research attention focusing on HIV risk reduction and management. Advances in antiretroviral therapy (ART) have led to higher survival and life expectancy rates in people living with HIV/AIDS [11, 12], who are now developing non-HIV related chronic conditions at higher rates than the general population. Several reviews suggest that both HIV and its treatment with antiretroviral therapy can accelerate the risks for chronic diseases associated with aging, particularly cardiovascular diseases and diabetes mellitus [13–15]. Physical activity is associated with reduced morbidity and mortality from chronic diseases [16–19], including heart disease [20, 21], stroke [22, 23], and diabetes mellitus [24, 25]. However, research has largely neglected the risk of these diseases among African American MSM and other sexual minorities [26].

Despite the well-established benefits of physical activity for a range of NCDs, it is estimated that only 28% of African American men meet the 2008 federal guidelines [27] for aerobic and muscle-strengthening physical activity. Maintaining engagement in physical activity behaviors is essential for reducing African American men’s long-term risk for NCDs and is especially beneficial for those living with HIV to help manage their disease [28, 29]. To date, only one physical activity intervention showed efficacy in increasing physical activity among African American MSM [26]. Understanding the psychosocial factors that influence African American men’s and particularly MSM’s physical activity is important to developing effective physical activity interventions for them.

### Syndemic theory

A growing literature centers on the syndemic theory, the notion that multiple adverse conditions interact synergistically, contributing to excess morbidity within a population [30, 31]. Conditions considered in the syndemic model usually include substance abuse, violence, childhood sexual abuse, and depression [32–34]. Studies have tied higher numbers of syndemic conditions to sexual risk behaviors and HIV infection among MSM [35, 36], management of chronic diseases among HIV-positive MSM [37], drinking patterns among Latino MSM and transwomen [38], and diabetes in low- and middle-income countries [37, 39]. Although some syndemic conditions, for instance, depression and anxiety have been studied in relation to physical activity [40–42], no study has examined whether the number of syndemic conditions is related to physical activity in any population.

A key premise of the syndemic theory is that syndemic conditions interact synergistically to affect health. Although a growing literature documents the relation of a greater number of syndemic conditions to health-related outcomes, recently Tsai and colleagues [43, 44] have challenged the traditional approach of testing syndemic conditions as cumulative adversities by using the sum of the number of syndemic conditions. They argue that studies have not actually tested synergistic effects, which would require testing interactions among the syndemic conditions in affecting outcomes. Finding that models including such interaction terms accounted for more variance than models without the interaction terms would be evidence of synergy among syndemic conditions.

### The present study

Beyond examining the relation of syndemic conditions to physical activity, the question of whether there are resilience factors that can potentially contribute to African American MSM’s physical health is also important and has received little empirical attention. Resilience factors have been studied in the context of HIV risks. Previous studies provide evidence suggesting that internal homophobia management, optimism, religiosity, cultural identity, social network support, and connection to a sexual minority community as potential sources of resilience [45–49]. In one sample of African American MSM, O’Leary and colleagues identified that optimism buffered the syndemic effect on HIV prevalence [32]. On physical activity, previous research has identified the influences of some sociodemographic factors. For instance, a higher level of income has been tied to more physical activity in African American men [50]. Studies have also linked social network diversity to more physical activity [51–53].

Building on previous studies, this secondary data analysis examined the relations of syndemic conditions and resilience factors to physical activity among African American MSM, participants in a 12-month health-promotion intervention trial [26, 54]. We tested two primary hypotheses: that the number of syndemic conditions reported pre-intervention would predict less self-reported physical activity 6 and 12 months post-intervention, adjusting for intervention condition, and that, consistent with the syndemic theory, syndemic conditions would interact to predict less physical activity, indicating a synergic effect. In addition, exploratory analyses examined whether participants’ physical activity levels differed by their HIV status, and whether potential resilience factors tested in previous research, including connection to the gay community, religiosity, black pride, optimism, and social network diversity, would predict higher levels of physical activity. Moreover, we examined whether these resilience factors buffered the relations of the syndemic conditions. Finally, we examined the relations of sociodemographic factors including age, education, and income to physical activity because previous research suggest they may be associated with physical activity [55, 56].

## Methods

This article reports findings from a longitudinal survey collected as a part of a behavioral intervention, “Project Being Responsible for Ourselves” (BRO), designed to reduce health risks including cardiovascular diseases, cancers, and STIs, including HIV for African American MSM. The trial randomized 595 African American MSM to either a physical-activity intervention or a HIV/STI risk-reduction intervention and assessed a variety of health outcomes including physical activity 6- and 12-months post-intervention. Details of the trial’s methods are reported elsewhere [26, 54]. Institutional review boards at the University of Pennsylvania and Temple University approved the study. We recruited participants in the Philadelphia area through advertising in local newspapers, through community-based organizations, through flyers posted at colleges, universities, bars, adult bookstores, through face-to-face recruitment at social events expecting a high African American MSM turnout, and through the referrals of participants. The recruitment materials explained that the research was designed to reduce health risks, including cardiovascular diseases, cancers, and STIs, including HIV. The eligibility criteria were based on the HIV/STI risk-reduction intervention, which was the primary intervention the study was funded to test. Men were eligible if they were at least 18 years of age, self-identified as black or African American, were born a male, and reported having anal intercourse with a man in the past 90 days. Men were excluded if they reported having anal intercourse with only 1 main male partner in the past 90 days. Informed consent was required for participation.

Participants were enrolled between April 2008 and March 2011, with all data collection completed by May 2012. Participants completed pre-intervention, post-intervention, and 6 and 12 months post-intervention questionnaires. The intervention and data-collection sessions were implemented at a university research center. The original trial involved 595 African American MSM: 295 in the HIV/STI risk-reduction and 300 in the physical-activity intervention. Among the 595, 168 said they were HIV-positive at the pre-intervention assessment.

### Measures

The participants completed confidential questionnaires via audio computer-assisted self-interviewing (ACASI) technology, providing audio and video presentation of the questions and response options on a laptop. ACASI has been shown to increase reports of socially undesirable behaviors compared with face-to-face interviews and self-administered surveys, possibly reflecting more accurate responding [57]. Psychosocial and physical activity variables were assessed pre-intervention and 6 and 12 months post-intervention.

#### Physical activity

Physical activity was assessed with 3 items the Centers for Disease Control and Prevention developed concerning the number of days on which people did vigorous-intensity aerobic activities for at least 20 min, moderate-intensity aerobic activities for at least 30 min, and strength-building activities in the past 7 days [58]. The outcome was a weighted average of the number of days on which participants reported engaging in 20 minutes of vigorous-intensity activity, 30 min of moderate-intensity activity, and strength-building activity, in the past 7 days. The 2008 physical activity guideline [27] requires 20 min of vigorous-intensity activity on at least 4 days or 30 min of moderate-intensity activity on at least 5 days and engaging in strength-building activity on 2 or more days, in the past 7 days. Accordingly, the weighted average was calculated as a sum of the weighted days, as follows: (days of vigorous activity/4 + days of moderate activity/5 + days of strength building activity/2) / (1/4 + 1/5 + 1/2). This formula differed from the original formula used in the main intervention trial [26], but the two formulas yielded highly correlated variables (*r* = .97 for the two baseline physical activity measurements, p < .0001).

#### Education

Participants reported their education level by answering the question “What is the highest grade of school you have completed?” We created a binary variable of having received at least a high school diploma (or GED) versus having received less than a high school education.

#### Income

Participants reported their monthly income in seven categories that ranged from “less than $400” to “1651 and above.” We created a categorical variable with 3 categories: “Less than $400,” “$400 - $850,” and “$851 or more.”

#### Syndemic condition

Syndemic condition is a composite variable summing 6 binary syndemic factors including depression [59], childhood sexual abuse [60], alcohol dependency [61], drug dependency [62], intimate partner violence [63], and unemployment [43]. We constructed the variable, as in previous studies [32], by adding the number of syndemic conditions experienced by each man. The syndemic condition variable thus ranged from 0 to 6.

Resilience factors include the following measures.

#### Connection to the gay community

Participants responded “yes” or “no” to the question, “Do you consider yourself to be a member of the gay community?” as used in previous research [46].

#### Religiosity

Participants reported the frequency with which they engage in specific religious activities, on a scale from 1 (never) to 5 (twice a week or more), as used in previous research [64]. Questions included “(How often do you) go to church or worship services,” “read the Bible or other religious works,” and “listen to religious radio stations?” The sum of endorsed items became the scale score.

#### Black pride

Black pride was assessed with a 7-item Likert scale validated in previous research [65]. Responses ranged from 1 (disagree strongly) to 5 (agree strongly). Example items included “I believe that because I am black, I have many strengths” “I feel that blacks have made major accomplishments and advancements” and “I feel good about black people.” The item average became the scale score (*a* = 0.82).

#### Optimism

This was assessed with Cantril’s Self-Anchoring Ladder scale [66, 67], which was comprised of two items with values ranging from 1 (worst possible life) to 10 (best possible life). Respondents indicated the level at which they placed themselves “at the present time” and “one year from now.” Optimism scores were computed by subtracting the “present” value from the “one year from now” value.

#### Social network diversity

Social network diversity was assessed with a 10-item scale validated in previous research [68], asking which of a number of different types of people to whom the respondent had spoken on the phone or in person in the previous 2 weeks. These included, for example, “spouse or steady partner,” “any of your children,” and “members of your church, mosque, etc.” The sum of endorsed items became the scale score.

### Statistical analysis

Descriptive statistics, including percentages, means, and standard deviations were used to describe the sociodemographic characteristics of the sample. T-test was conducted to examine the difference on physical activity between HIV positive and negative men. The bivariate relation of each baseline predictor (i.e., age, education, income, syndemic conditions, alcohol dependency, drug dependency, depression, childhood sexual abuse, intimate partner violence, unemployment, connection to the gay community, religiosity, black pride, optimism, social network diversity) to physical activity was tested using generalized-estimating-equations (GEE) models [69, 70], adjusting for longitudinal repeated measurements. The models included the baseline predictor, follow-up time (2 categories representing 6- and 12-month follow-up), and the intervention condition. To test the moderation effect of each resilience factor on the bivariate relation between syndemic conditions and physical activity, we added the syndemic conditions × resilience factor interaction term in the GEE models.

In addition to the bivariate relations, we tested one complete GEE model (model 1) that included the demographic predictors, syndemic conditions, and all resilient factors. The model included 9 baseline predictors, follow-up time (2 categories representing 6- and 12-month follow-up), and the intervention condition. Lastly, we tested an alternative complete GEE model (model 2) that included the demographic predictors, all 6 individual syndemic factors, and all resilient factors. We used robust standard errors and specified an independent working correlation matrix for all the GEE models. We report regression coefficients and their corresponding 95% confidence intervals.

To test whether individual syndemic factors had synergistic effects on predicting physical activity, we conducted explorative tests to identify all potential interaction effects among the 6 syndemic factors. Specifically, we tested 15 GEE models for all 2-way interactions, 20 models for all 3-way interactions, 15 models for 4-way interactions, 6 models for 5-way interactions, and 1 model for the 6-way interaction. These models included only the interested syndemic factors and their interactions, adjusting for time and intervention condition. We applied Bonferroni correction for each set of interaction tests and adjusted the significance criteria for the 2-way, 3-way, 4-way, 5-way, and 6-way interactions to be .003, .0025, .003, .008, and .05. After we identified a significant interaction effect, we then compared the R-squared values of the interaction model with its corresponding baseline model without the interaction terms to determine whether adding the interactions improved the model fit in predicting physical activity. All analyses were performed using SAS V9.3.

## Results

The participants were 595 African American MSM. Participants’ age ranged from 18 to 69 years (mean = 41.6; SD = 10.7). As shown in Table 1, only 28.5% were employed and 48.4% had completed high school. About 72.7% reported their monthly income was less than $851. About 40.6% self-identified as gay, 41.3% self-identified as bisexual, 10.5% said they were on the down low [71], and 7.6% self-identified as straight. The majority (96.0%) of participants had ever tested for HIV and 29.5% said they were HIV-positive. About 44.5% were alcohol dependent, 16.7% were drug dependent, and 51.8% had spent time in jail or prison (mean = 1071.8 days; SD = 1637.0). Importantly, 74.4% experienced at least one of the syndemic conditions.

**Table 1:** Baseline Socio-demographic characteristics of participating African American men, Philadelphia, PA, 2008–2011. Some of the data has been published in the original intervention trial reports [26, 54].

Characteristic	Total
No.	595
Age, mean (SD)	41.6 (10.7)
Employed	169/593 (28.5%)
Completed high school	287/593 (48.4%)
Monthly income	
Less than $ 400	219/593 (36.9%)
$ 400 - $ 850	212/593 (35.8%)
$ 851 or more	162/593 (27.3%)
Had stable housing	463/593 (78.1%)
Sexual self-identity	
Gay	241/593 (40.6%)
Straight	45/593 (7.6%)
Bisexual	245/593 (41.3%)
On the down low ^a^	62/593 (10.5%)
Ever tested for HIV	569/593 (96.0%)
HIV positive	168/569 (29.5%)
Ever in jail or prison	307/593 (51.8%)
Days in jail or prison	1071.8 (1637.0)
Syndemic factors	
Alcohol dependency ^b^	264/593 (44.5%)
Drug dependency ^c^	99/593 (16.7%)
Depression	112/593 (20.6%)
Childhood sexual abuse	290/593 (48.9%)
Intimate partner violence	220/593 (37.1%)
Unemployment	424/593 (71.5%)
Syndemic conditions	
0	152/593 (25.6%)
1	174/593 (29.3%)
2	145/593 (24.5%)
> = 3	122/593 (20.6%)
Resilient factors	
Connection to the gay community	204/593 (34.4%)
Religiosity, mean (SD)	2.89 (1.05)
Black pride, mean (SD)	4.20 (0.65)
Optimism, mean (SD)	0.49 (2.48)
Social network diversity, mean (SD)	4.36 (2.57)

Notes: ^a^ “on the down-low” is a term describing African American men who have sex with men who do not identify as gay or disclose their bisexual activities to their main female partners.

^b^ Based on a score of 2 or greater on the CAGE (Cutting down, Annoyance by criticism, Guilty feeling, and Eye-openers) questionnaire.

^c^ Based on a score of 3 or greater on the TCUDS (Texas Christian University Drug Screen) questionnaire.

**Fig 1:**
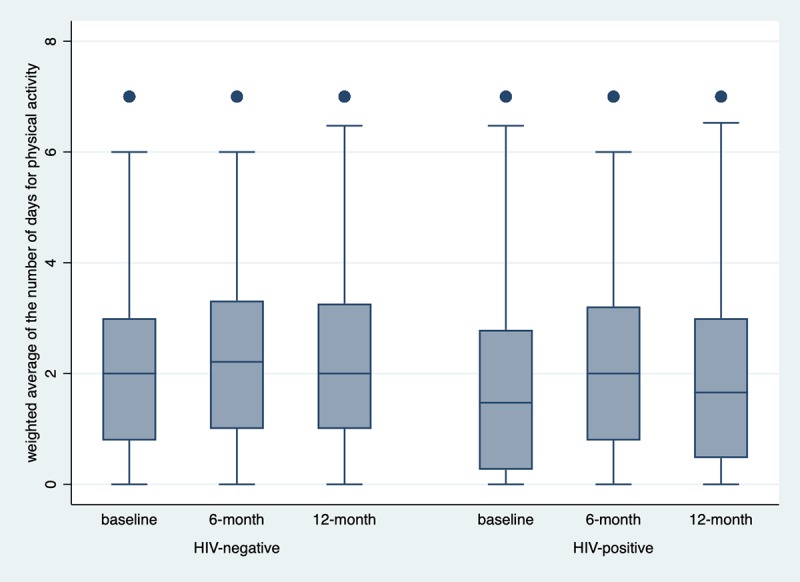
Distributions of the weighted average days for physical activity by HIV status across time.

Fig 1 depicts the medians and ranges of the weighted average days for physical activity by participants’ HIV status across the three assessment periods.

Table 2 shows participants’ self-reported days for different types of physical activities by their HIV status. Participants’ weighted average days for physical activity differed by their HIV status at baseline, but not at the follow-up assessment periods. At baseline, only 118 (19.9%) participants met the 2008 national physical activity guideline. HIV-negative men reported an average of 2.14 days (SD = 1.71) for physical activity whereas HIV-positive men reported an average of 1.82 (SD = 1.77) (p = 0.044). This suggests HIV positive men engaged in less physical activity compared to HIV negative men. However, they might respond well to the intervention and the follow-up assessments did not show a difference by HIV status.

**Table 2:** Days for physical activity by HIV status and assessment period, African American men, Philadelphia, PA, 2008–2011.

	HIV negative mean (SD)	HIV positive mean (SD)	P value of t-test
**Baseline**			
Weighted average days for physical activity	2.14 (1.71)	1.82 (1.77)	0.044
Days for vigorous activity	2.22 (1.99)	2.05 (2.18)	0.385
Days for moderate activity	2.67 (2.29)	2.45 (2.46)	0.308
Days for strength building activity	1.89 (1.98)	1.45 (1.91)	0.015
**6-month follow-up**			
Weighted average days for physical activity	2.39 (1.77)	2.16 (1.82)	0.190
Days for vigorous activity	2.74 (2.04)	2.38 (2.14)	0.073
Days for moderate activity	2.81 (2.20)	2.62 (2.26)	0.379
Days for strength building activity	2.04 (1.99)	1.86 (2.00)	0.365
**12-month follow-up**			
Weighted average days for physical activity	2.34 (1.78)	2.01 (1.82)	0.059
Days for vigorous activity	2.52 (1.99)	2.28 (2.10)	0.217
Days for moderate activity	2.77 (2.04)	2.53 (2.32)	0.248
Days for strength building activity	2.08 (1.93)	1.67 (1.93)	0.030

Regarding prospective predictors, as shown in Table 3, in the bivariate regression models, age, education, syndemic conditions, drug dependency, depression, intimate partner violence, and resilience factors including black pride and social network diversity significantly predicted physical activity averaged over the 6- and 12-month follow-up assessments. The complete models 1 and 2 show similar results, except that black pride was no longer a significant predictor. Specifically, being older and experiencing more syndemic conditions predicted less physical activity. Receiving high school education and having greater levels of social network diversity predicted more physical activity.

**Table 3:** GEE Empirical significance tests for the relations of baseline socio-demographic characteristics to physical activity averaged over the 6- and 12-month follow-up assessments, African American men, Philadelphia, PA, 2008–2011.

Baseline predictor	Bivariate model coefficient (95% CI)	Complete model 1 coefficient (95% CI)	Complete model 2 coefficient (95% CI)
Age	-0.02 (-0.03, -0.003)*	-0.02 (-0.03, -0.002)*	-0.02 (-0.03, -0.002)*
Education	0.44 (0.08, 0.81)*	0.37 (0.01, 0.72)*	0.38 (0.02, 0.74)*
Income	-0.01 (-0.14, 0.11)	-0.07 (-0.19, 0.06)	-0.06 (-0.19, 0.08)
Syndemic conditions	-0.15 (-0.24, -0.06)***	-0.17 (-0.27, -0.08)***	
Alcohol dependency	-0.17 (-0.47, 0.12)		-0.03 (-0.33, 0.26)
Drug dependency	-0.40 (-0.74, -0.06)*		-0.23 (-0.59, 0.14)*
Depression	-0.41 (-0.72, -0.10)**		-0.31 (-0.66, 0.03)**
Childhood sexual abuse	-0.18 (-0.45, 0.08)		-0.18 (-0.45, 0.08)
Intimate partner violence	-0.28 (-0.55, -0.02)*		-0.17 (-0.43, 0.10)*
Unemployment	-0.10 (-0.39, 0.19)		-0.12 (-0.41, 0.10)
Gay community connection	0.01 (-0.27, 0.29)	-0.002 (-0.27, 0.27)	-0.001 (-0.26, 0.26)
Religiosity	0.13 (-0.01, 0.26)	0.08 (-0.07, 0.22)	0.08 (-0.07, 0.22)
Black pride	0.27 (0.07, 0.48)**	0.13 (-0.08, 0.34)	0.12 (-0.10, 0.33)
Optimism	0.04 (-0.01, 0.10)	0.04 (-0.01, 0.09)	0.04 (-0.01, 0.09)
Social network diversity	0.12 (0.06, 0.18)***	0.10 (0.04, 0.16)***	0.10 (0.04, 0.16)***

Note.

*p<0.05

**p<0.01

***p<0.001

We did not find any evidence of a moderating role of any resilience factor on the relations between syndemic conditions and physical activity. In other words, the negative relation of syndemic conditions to physical activity did not differ for different levels of gay community connection (p = 0.123), religiosity (p = 0.885), black pride (p = 0.417), optimism (p = 0.993), or social network diversity (p = 0.411).

Using the adjusted significance criteria for all tested interaction models, we did not find any significant interaction effect of the syndemic conditions in predicting physical activity.

## Discussion

To our knowledge, this is the first study to provide empirical data on the relations of syndemic conditions and levels of physical activity and the psychosocial predictors among African American MSM. The results contribute to the limited research on physical activity among MSM in general, and among African American MSM specifically. The sample of 595 African American MSM experienced a composite of social and economic disadvantages. Less than one-third were employed, one-third were living with HIV, and one-half had spent time in jail or prison. The majority had experienced at least one of the syndemic conditions.

Previous research documented that people living with HIV engaged in less physical activity than most other populations with chronic diseases [72, 73]. However, one study conducted in Australia showed that HIV-positive participants reported higher levels of physical activity than the HIV-negative group [74]. In our sample, we observed HIV positive participants engaged in less physical activity than HIV negative participants at baseline. However, the difference disappeared after the intervention in the following year. Nonetheless, both groups engaged in less than 2.5 weighted days of physical activity per week, suggesting the necessity to introduce physical activity intervention programs for this population to mitigate the long-term risks for NCDs.

As we hypothesized, this research identified a negative association of syndemic conditions to physical activity. Our longitudinal survey data showed robust evidence that the number of syndemic conditions reported at baseline directly predicted lower levels of physical activity during the following year, indicating a potential additive influence of the negative psychosocial experiences of depression, childhood sexual abuse, alcohol problems, problems with drug use, intimate partner violence, and unemployment. The more conditions participants experienced, the less physical activity they engaged in the following year. Although previous research documented interactions of some of these factors in impacting HIV risks [43], we did not find any synergistic interaction effect among these syndemic factors in predicting physical activity.

On resilience factors, we found social network diversity prospectively predicted higher levels of physical activity. This finding corroborated one survey study which concluded higher network diversity was associated with an increased likelihood of physical activity [53]. This finding is in line with other observations showing a positive relationship between social connections and healthy lifestyle [75].

There are limitations to this study that should be considered when interpreting results. The data are from African American men who lived in Philadelphia, Pennsylvania; therefore, the results may not be representative of other African American MSM populations. The physical activity behaviors were self-reported, therefore subjective to recall bias. Notwithstanding these limitations this is the first study to provide information on physical activity among African American HIV-positive and HIV-negative MSM, a minority group within a minority population characterized by disproportional health problems. The prospective design is an additional strength, which allows for establishing temporal precedence in investigating the relations between psychosocial factors and physical activity. However, it is important to note that the predictive relations do not indicate causality. While factors such as depression and substance abuse can cause negative health outcomes, we cannot rule out the possibility that a lack of physical activity may also contribute to depression and substance abuse.

The findings from this study suggest important directions for developing physical activity promotion interventions for this population. The same types of interventions can be used to promote physical activity among African American MSM regardless of their HIV status. Intervention contents should address the syndemic conditions experienced by African American MSM. For instance, interventions can consider addressing depression, alcohol and drug use, and sexual violence alongside improving physical activity. Lastly, given the significant association of social network diversity to physical activity, social network-level interventions that involve building and expanding supportive networks of African American MSM should receive more attention and empirical test in the future to enhance the general physical health of this population.
